# Evaluating the Efficacy of Xylitol Wipes on Cariogenic Bacteria in 19- to 35-month-old Children: A Double-blind Randomized Controlled Trial

**DOI:** 10.5005/jp-journals-10005-1476

**Published:** 2017-02-01

**Authors:** Gurusamy Kayalvizhi, Dhayalan Nivedha, Renganathan Sajeev, Gajula S Prathima, Mohandoss Suganya, Venkatesan Ramesh

**Affiliations:** 1 Professor, Department of Pedodontics and Preventive Dentistry, Indira Gandhi Institute of Dental Sciences, Puducherry, India; 2 Postgraduate Student, Department of Pedodontics and Preventive Dentistry, Indira Gandhi Institute of Dental Sciences, Puducherry, India; 3 Professor, Department of Pedodontics and Preventive Dentistry, Indira Gandhi Institute of Dental Sciences, Puducherry, India; 4 Professor, Department of Pedodontics and Preventive Dentistry, Indira Gandhi Institute of Dental Sciences, Puducherry, India; 5 Senior Lecturer, Department of Pedodontics and Preventive Dentistry, Indira Gandhi Institute of Dental Sciences, Puducherry, India; 6 Senior Lecturer, Department of Pedodontics and Preventive Dentistry, Indira Gandhi Institute of Dental Sciences, Puducherry, India

**Keywords:** Bacteria, Cariogenic, Child daycare center, Prevention, *Streptococcus mutans*, Xylitol.

## Abstract

**Introduction:**

Dental caries is an infectious disease with *Streptococcus mutans* as the main cariogenic bacteria. Children with early S. *mutans* colonization have a higher risk of developing dental caries than those with later colonization. Therefore, prevention or delay of S. *mutans* colonization may be advantageous for the prevention of early childhood caries (ECC).

**Aim:**

To evaluate and compare the effectiveness of xylitol and placebo wipes on S. *mutans* count in 19- to 35-month-old children.

**Materials and methods:**

Forty-four children were randomly selected from a daycare center and divided into two groups. Allocation concealment was done and both (placebo and xylitol) wipes were distributed to their parents. Instructions were given regarding their use, to be used twice daily for 2 weeks and the S. *mutans* levels in the saliva were enumerated before and after wipes usage. The collected data were tabulated and statistically analyzed using paired and unpaired t-tests.

**Results:**

A clinically significant decrease in the S. *mutans* count was observed in the xylitol wipes group than the placebo wipes group. Intergroup comparison results were found to be statistically insignificant.

**Conclusion:**

Xylitol wipes usage could serve as a useful adjunct in reducing the cariogenic bacteria, especially *S. mutans,* and thus can be considered as an adjunct oral hygiene tool for caries prevention in young children

**How to cite this article:** Kayalvizhi G, Nivedha D, Sajeev R, Prathima GS, Suganya M, Ramesh V. Evaluating the Efficacy of Xylitol Wipes on Cariogenic Bacteria in 19- to 35-month-old Children: A Double-blind Randomized Controlled Trial. Int J Clin Pediatr Dent 2018;11(1):13-17.

## INTRODUCTION

Early childhood caries is a chronic infectious disease with *S. mutans* being the main cariogenic bacteria in humans. Children with early *S. mutans* colonization have a higher risk of developing caries than those with later colonization. Therefore, prevention or delay of *S. mutans* colonization may be advantageous for the prevention of ECC.^1^

Xylitol is a sugar substitute with sweetness equal to that of sucrose, but with 40% fewer calories. It has been found in small quantities in fruits and vegetables and is produced as a part of human metabolic process.^2^ It decreases the synthesis of insoluble extracellular poly-saccharides *in vitro,* thus habitual xylitol consumption might limit adhesion of *S. mutans* to the enamel, thereby inhibiting their virulence.^1^

Xylitol has been used in various forms like chewing gum, mints, energy bar foods, nasal sprays, oral hygiene products (e.g., mouth rinse, syrups, gels, wipes, and floss) and found to be effective in caries prevention.^3^ Le and Caufield^4^ stated that the direct use of xylitol products in young children remains a potentially effective regimen than its maternal use to block *S. mutans* transmission and to prevent caries in children. In 2008, the American Academy of Pediatric Dentistry recommended tooth wipes as an important tool for oral hygiene care in infants and toddlers. The xylitol wipe usage has been shown to be safe and well accepted by both the parents and infants.^1^

Studies^1,5^ have found that mothers can significantly reduce caries in their babies by wiping their mouths with wipes impregnated with xylitol. However, till date, there are no published studies assessing the effect of xylitol wipe use in reducing *S. mutans* acquisition in young children in India. Thus, the aim of the present study was to evaluate the effect of xylitol wipes on *S. mutans* in Panruti, Cuddalore district, Tamil Nadu, India.

## MATERIALS AND METHODS

### Study Population and Study Design

A double-blind randomized controlled trial was conducted after obtaining approval from the ethical committee. Sample size calculation was done based on previous literature and using the sample size formula.^1^ Allocation concealment was done by sequentially numbered, opaque, sealed envelopes. Children with oral or systemic diseases and who were on antibiotics or other medications in the past 3 months that would affect the oral flora were excluded. After obtaining permission from the daycare center and consent from the parents, 44 children were randomly selected in the age group of 19 to 35 months by the toss of a coin. The placebo and xylitol wipes were assigned randomly to these children. Both the groups were blinded.

**Fig. 1:**
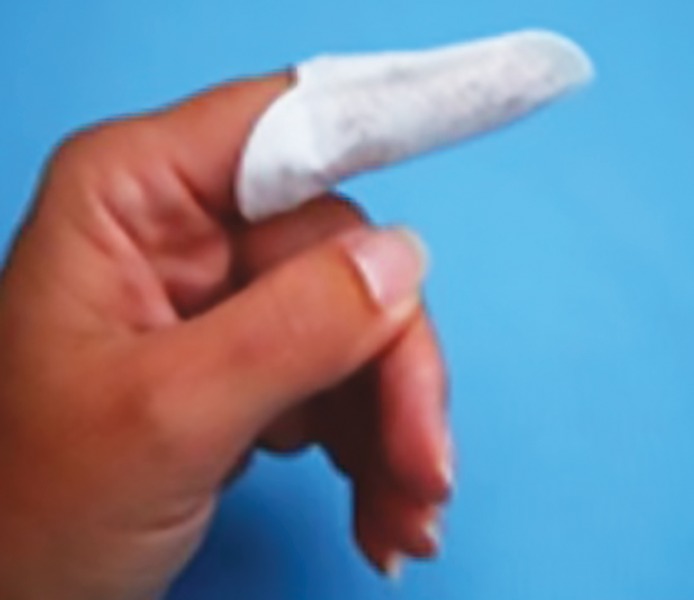
Wipes wrapped on index finger

**Fig. 2:**
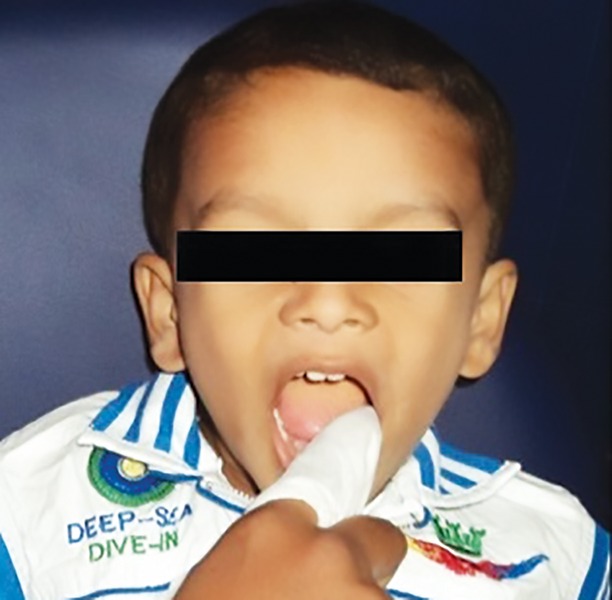
Procedure of wipes usage

Wipes were prepackaged for distribution in daily bags (two doses per bag) labeled with the day of the week, and placed in larger bags labeled with the identification number 1 and 2. An independent examiner who was not part of the study packed the wipes and labeled it as 1 and 2 and wrote 1 and 2 belonged to which wipes and placed it in a sealed cover, which was given to the coinvestigator. The wipes numbered as 1 and 2 were randomly distributed to the mothers for their use in children. Both the principal and coinvestigators were also blinded. The placebo wipes were identical in appearance and composition, except that there was no xylitol in the placebo wipe. Both wipes were apple flavored, which were procured from Spiffies Baby Tooth Wipes™ (DR Products Inc., USA). The wipes were provided to the mother and they were instructed to open the packet, unfold the wet wipe, wrap around the finger (Fig. 1), and wipe over the baby’s teeth (Fig. 2). They were advised to use them twice daily 1 hour after food to clean the teeth and gums of the children, in addition to toothbrushing for 14 days.

### Saliva Collection

Before sample collection, children were advised to rinse their mouth with drinking water and to wait at least 5 minutes before providing a saliva sample. Unstimulated saliva was collected by spitting method; the children were advised to spit into the disposable cup up to the level indicated on the collection container (approximately 2 mL) which was then transferred to the tube. All samples were collected under the supervision of trained personnel, as subjects were typically too young to follow the instructions.

Mothers were telephoned by the principal investigator over the phone once in 2 days to check for compliance and side effects of wipe usage, including allergy, gastritis, and diarrhea. The daily usage was monitored by the coinvestigator. Unstimulated saliva was collected from the children before the usage of wipes and after 14 days. The collected saliva samples were transported on thio-glycolate medium to the laboratory for bacterial culture.

### Microbial Assays

The saliva samples were cultured on mitis salivarius sucrose bacitracin agar for *S. mutans.* The plates were incubated anaerobically (85% N_2_, 5% CO_2_, and 10% H_2_) at 37°C for 72 hours for subsequent enumeration of *S. mutans* colonies with the use of a dissecting microscope. The colony-forming units per milliliter were evaluated.

The obtained data were tabulated and subjected to statistical analysis.

### Statistical Analysis

Descriptive statistics, such as mean, standard deviation, and standard error of the mean were used to summarize the data. Paired t-tests were performed using a 0.05 significance level to determine if significant differences existed within the two groups. Unpaired t-tests were performed between the xylitol and placebo groups.

## RESULTS

The major objective of the study was to find out any difference in the *S. mutans* count before and after wipes usage and between xylitol wipes and placebo wipes groups. We had no dropouts, as it was a 14-day trial. No reported side effects were observed.

**Table 1:** **Table 1:** Difference in the S. *mutans* count before and after 14 days of placebo wipe usage

				*Paired differences*											
				*Mean*		*Std. deviation*		*Std. error mean*		*t-value*		*Degree of freedom*		*Signification (2-tailed)*	
Pair 1		Before and after wipes usage		0.440		4.564		1.021		0.432		19		0.671	

**Table 2:** **Table 2:** Difference in the S. *mutans* count before and 14 days after xylitol wipes usage

		*Paired differences*											
		*Mean*		*Std. deviation*		*Std. error mean*		*t-value*		*Degree of freedom*		*Significance (2-tailed)*	
Before and after xylitol wipes usage		1.531		6.105		1.365		1.121		19		0.276	

**Table 3:** **Table 3:** Comparison between the placebo and xylitol wipe groups using unpaired t-test

		*Group*		*n*		*Mean*		*Std. deviation*		*Std. error mean*		*Significance*	
Before-wipe usage		Placebo		22		2.35		3.198		0.715		0.479	
		Xylitol		22		3.24		4.903		1.096			
After-wipe usage		Placebo		22		1.91		2.930		0.655		0.839	
		Xylitol		22		1.71		3.541		0.792			

### Paired t-test for Placebo Group

It was found that the mean difference between before and after the placebo wipes usage was 0.44 ± 4.56. When 2-tailed tests were applied, a p-value of 0.67 was obtained, which was statistically insignificant. These results state that no significant reduction in the number of *S. mutans* after 14 days of placebo wipes usage was observed (Table 1).

### Paired t-test for Xylitol Wipes Group

It was found that the mean difference before and after the xylitol wipes usage was 1.53 ± 6.11, while with 2-tailed test, a p-value of 0.28 was obtained, which was also not significant statistically. In the xylitol group, also the reduction in the number of *S. mutans* was observed, but it was not significant (Table 2).

### Unpaired t-test between the Two Groups

When unpaired t-test was used to compare the *S. mutans* count between the two groups before and after wipes usage, it was found that before wipes usage, both groups had a p-value of 0.479, and after wipes showed a p-value of 0.839, although there was no statistically significant (value) decrease in the *S. mutans* count between groups. We found the reduction to be more in the xylitol group when compared with the placebo group (Table 3).

## DISCUSSION

Dental caries is a common chronic infectious transmissible disease resulting from tooth-adherent specific bacteria, primarily mutans streptococci (MS) that metabolizes sugars to produce acid which, over time, demineralizes the tooth structure. The disease of ECC is the presence of one or more decayed (noncavitated or cavitated lesions), missing (due to caries), or filled tooth surfaces in any primary tooth in a child under the age of 6. In children younger than 3 years, any sign of smooth-surface caries is indicative of severe ECC.^6^

Xylitol is a sugar substitute with sweetness equal to that of table sugar (sucrose), but with 40% fewer calories. It has been approved by the Food and Drug Administration since the 1960s and is safe for use in children. In addition, xylitol has a number of other effects on *S. mutans* that may account for some of its clinical effects in caries reduction. Short-term consumption of xylitol products is associated with decreased *S. mutans* levels in both saliva and plaque.^5^ So, this study was planned to evaluate the efficacy of xylitol wipes in reducing the *S. mutans* count in children with ECC.^7^

Informed consent was obtained from the parents on parent-teacher’s meet, such that it was easy to explain the parents about the need for oral hygiene in infants and toddlers and instructions were given regarding the use of the wipes.

The sample size was selected based on the sample size formula and Zhan’s study,^1^ which also compared xylitol with placebo wipes similar to our study. The present study was conducted in Panruti, Cuddalore District, to detect a difference at alpha (two-sided = 0.05, power = 80%) an effect size of 38.8% was estimated as 22 children per group. They were randomly allocated to xylitol and placebo wipes groups. In contrast, Almeida et al^5^ conducted a randomized crossover trial using xylitol wipes and manual toothbrush as control in children.

In 1993, Li and Caufield^4^ described a discrete “window of infectivity” during which infants acquired MS from their maternal host. This Ȍwindow” opened at 19 months and extended to 31 months, with a mean of 26 months. During this period, the prevalence of MS was seen to rise from 0 to 82% empirically; if the acquisition of these cariogenic bacteria could be blocked during this period, an individual may never acquire these organisms and thereby experience lifelong immunity from dental caries. A study showed that 83% of children had an MS colonization by 36 months, and that this group of children would be ideal for implementing tooth wipes as an adjunct oral hygiene tool.^4^ Thus, in the present study, children aged 19 to 35 months were selected. On the contrary, Zhan et al study assessed the streptococci and lactobacilli salivary counts in the age group of 6- to 35-month-old children,^1^ while Almeida et al^5^ study assessed the plaque removal efficacy of xylitol tooth wipes in high-caries-risk 8- to 15-month-old babies before primary molar eruption. Milgrom et al conducted their study in 15 to 25 months aged children, but they used xylitol syrup instead of wipes during primary tooth eruption in children and found reduction in tooth decay.^7^

Both the investigators and the parents were blinded to which group the participants belonged. An independent examiner who was not part of the study received the wipes, sealed it, and numbered it as 1 and 2. To overcome bias, we used apple-flavored wipes (Dr Spiffie’s wipes) in both groups. The coinvestigator was appointed to monitor the daily wipes usage. In this study, they were instructed to use xylitol wipes twice daily for 14 days, which delivered 2.6 gm of xylitol which is less than the normal minimal level of 3 gm/day which may be the reason for not obtaining a significant reduction in *S. mutans* count. Whereas in Zhan et al^1^ study, they used 4 wipes twice daily for 1 year which provided 4.2 gm/ day and in Almeida study,^5^ they used the wipes only for 2 days. We introduced xylitol wipes slowly because according to the American Academy of Pediatric Dentistry, it should be used very slowly over a week to acclimate the body to the polyol, especially in young children, to avoid side effects like gas and diarrhea.^3^

In the present study, we assessed the *S. mutans* count as it is the major cariogenic organism in the initiation of caries, whereas Zhan et al^1^ study determined the reduction in *S. mutans* and lactobacilli and the number of carious lesions. Similar studies like that of Almeida et al^5^ evaluated the plaque removal efficacy of the xylitol wipes and Goval et al^8^ compared the sugar-free textured wipes with that of toothbrushing.

We found the reduction in the number of *S. mutans* to be more in the xylitol group than in the placebo group after wipes usage because the microorganisms do not readily metabolize xylitol into energy sources and its consumption has a minimal effect on plaque pH. However, it is absorbed and accumulates intracellularly in *S. mutans,* by competing with sucrose for its cell-wall transporter and intracellular metabolic processes. Unlike the metabolism of sucrose, which produces energy and promotes bacterial growth, *S. mutans* spends energy to break down the accumulated xylitol without yielding energy in return. Furthermore, the energy-producing intermediates are consumed and are not reproduced by xylitol metabolism. This has been demonstrated *in vitro,* which might contribute to the reduction of *S. mutans* levels in plaque and saliva and a reduction in acid production among those consuming xylitol.^9^

In this study, xylitol and placebo wipes were used to see if there was any decrease in the number of *S. mutans* counts after its short-term use. It was found that there existed a difference in the number of *S. mutans* count between the two groups, although no statistically significant difference was observed in their reduction. These results are in correlation with Zhan et al study who also found no statistically significant reduction in the *S. mutans* count between the two groups in the levels of *S. mutans* and *lactobacilli* at all time points. The MS levels remained stable from baseline to 6 months in both groups, but doubled or tripled at 1 year compared with baseline, resulting in a significant increase at 1 year compared with previous visits in both groups. They found that significantly fewer children in the xylitol wipe group had new caries lesions at 1 year compared with those in the placebo wipe group.^1^ Thus, our study findings support the anticaries efficacy with the direct use of xylitol in infants and children. We can assume that xylitol modified the virulence of cariogenic bacteria.

Another study^5^ assessed the plaque removal efficacy in high-caries-risk babies and found significant results in primary anterior teeth, with xylitol wipes as well as manual toothbrushing. However, their initial plaque index score before and after performance of cleaning methods was not significant, which is similar to our study results, but in contrast, we evaluated only *S. mutans* count using xylitol and placebo wipes. Our results are incomparable with this study, as the authors stated that their results could not be extrapolated to children after the eruption of primary molars.

Goval et al^8^ study used a novel sugar-free textured tooth wipes in adults which significantly reduced plaque levels from smooth surfaces in a randomized single crossover study. They found it to be safe and recommend them in cases where toothbrushing is impractical. These results coincide with the present study wherein placebo wipes also reduced the *S. mutans* count, which could be attributed to the mechanical action of wipes usage.

In the present study, we also compared within the groups before and after the wipes usage for both xylitol and placebo groups and found that there was a reduction in the number of *S. mutans* in both groups, although no statistical significance was found. It would have been because wipes do not effectively clean the primary molar pit and fissure surfaces which are the main sites of *S. mutans* colonization and its short-term usage.

We found a considerable reduction in the *S. mutans* count in the xylitol wipes group in our short-term trial (14 days) which could be clinically significant on a long-term use. In contrast, Zhan et al^1^ study was carried out for a year (at 3, 6, and 12 months follow-up) which also found no reduction in the *Lactobacillus acidophilus* and *S. mutans* count but decreased new caries incidence, whereas Almeida et al^5^ compared the plaque removal efficacy of xylitol wipes after 48 and 72 hours’ washout period in a crossover trial. Similarly, Goval et al^8^ evaluated the plaque removal efficacy of sugar-free wipes for 1 minute and evaluated after a 1-week washout period in adults and found it to be effective. The present study results are inconsistent with previous studies due to different follow-up protocols and varied parameters assessed.

Xylitol wipes were more accepted by both the parents and infants, especially at night^1,5^; we did not evaluate this fact in the form of a questionnaire, and even though we used the same flavored wipes, the sweetness in the xylitol wipes could act as a confounding factor compared with the placebo group.

Within the limitation of our study, xylitol wipes can be recommended as an adjunct to other toothbrushing modalities in children. It can be considered as an initial oral hygiene aid in caries risk infants from their first dental visit as anticipatory guidance. Further long-term studies are needed to evaluate the efficacy of xylitol wipes in reducing *S. mutans* counts and caries incidence, especially in children bottle-fed at night. It will be easier to wipe their teeth with wipes than brushing at nighttime to prevent ECC.

## CONCLUSION

We observed a greater decrease in the number of *S. mutans* counts with xylitol wipes usage when compared with the placebo group, which can be considered clinically significant. However, the reduction in *S. mutans* count before and after both wipes usage was not statistically significant.

## References

[1] Zhan L, Cheng J, Chang P, Ngo M, Besten PKD, Hoover CI, Featherstone JDB (2012). Effects of xylitol wipes on cariogenic bacteria and caries in young children. J Dent Res.

[2] Lynch H, Milogram P (2003). Xylitol and dental caries: an overview for clinicians. J Calif Dent Assoc.

[3] (2014). Guideline on Xylitol Use in Caries Prevention. Am Acad Pediatr Dent Ref Man.

[4] Li Y, Caufield PW (1995). The fidelity of initial acquisition of mutans streptococci by infants from their mothers. J Dent Res.

[5] Almeida AG, Queiroz MC, Leite AJM (2007). The effectiveness of a novel infant tooth wipe in high caries-risk babies 8 to 15 months old. Pediatr Dent.

[6] (2010). Policy on the use of xylitol in caries prevention. Am Acad Pediatr Dent Ref Man.

[7] Milgrom P, Kiet A, Ohnmar LY, Tut K, Mancl L, Roberts MC, Briand K, Gancio MJ (2009). Xylitol pediatric topical oral syrup to prevent dental caries: a double blind randomized clinical trial of efficacy. Arch Pediatr Adolesc Med.

[8] Goval CR, Qaqish JG, Sharma NC, Warren PR, Cugini M, Thompson MC (2005). Plaque removal efficacy of a novel tooth. J Clin Dent.

[9] Kiet A, Milgrom P, Rothen M (2006). Xylitol, sweeteners, and dental caries. Pediatr Dent.

